# AFM and Fluorescence Microscopy of Single Cells with Simultaneous Mechanical Stimulation via Electrically Stretchable Substrates

**DOI:** 10.3390/ma14154131

**Published:** 2021-07-24

**Authors:** Natalia Becerra, Barbara Salis, Mariateresa Tedesco, Susana Moreno Flores, Pasquale Vena, Roberto Raiteri

**Affiliations:** 1 Department of Informatics, Bioengineering, Robotics, and System Engineering (DIBRIS), University of Genova, 16145 Genova, Italy; natalia.becerra@gmail.com (N.B.); b.salis88@gmail.com (B.S.); brunella.tedesco@unige.it (M.T.); 2 Tissue Engineering and Cell Therapy Group (GITTC), School of Medicine University of Antioquia, Medellin 050010, Colombia; 3 Independent Researcher, 1190 Vienna, Austria; smf8097@gmail.com; 4 Department of Chemistry, Materials and Chemical Engineering Giulio Natta, Politecnico di Milano, 20133 Milan, Italy; pasquale.vena@polimi.it; 5 The National Research Council-Institute of Biophysics, 16149 Genova, Italy

**Keywords:** cell stretching, mechanical stimulation, dielectric elastomer actuators, atomic force microscopy, cellular biomechanics

## Abstract

We have developed a novel experimental set-up that simultaneously, (i) applies static and dynamic deformations to adherent cells in culture, (ii) allows the visualization of cells under fluorescence microscopy, and (iii) allows atomic force microscopy nanoindentation measurements of the mechanical properties of the cells. The cell stretcher device relies on a dielectric elastomer film that can be electro-actuated and acts as the cell culture substrate. The shape and position of the electrodes actuating the film can be controlled by design in order to obtain specific deformations across the cell culture chamber. By using optical markers we characterized the strain fields under different electrode configurations and applied potentials. The combined setup, which includes the cell stretcher device, an atomic force microscope, and an inverted optical microscope, can assess in situ and with sub-micron spatial resolution single cell topography and elasticity, as well as ion fluxes, during the application of static deformations. Proof of performance on fibroblasts shows a reproducible increase in the average cell elastic modulus as a response to applied uniaxial stretch of just 4%. Additionally, high resolution topography and elasticity maps on a single fibroblast can be acquired while the cell is deformed, providing evidence of long-term instrumental stability. This study provides a proof-of-concept of a novel platform that allows in situ and real time investigation of single cell mechano-transduction phenomena with sub-cellular spatial resolution.

## 1. Introduction

Mechanical stretch induces a wide range of cellular responses, including cytoskeletal remodeling, synthesis of extracellular matrix proteins, and altered expression of genes [1]. Cell reorientation is the most visible effect of stretching [2], and it is accompanied by a pronounced reorganization of the actin cytoskeleton [3], which can produce changes in cellular stiffness. The deformation-dependent increase in stiffness has been interpreted as an evidence of the nonlinear elastic response of actin cytoskeletal networks. This working hypothesis enabled an explanation of the observed significant changes in cell stiffness under stretched conditions that were not associated to alterations in the cytoskeletal assembly-disassembly.

Several laboratory-made devices have been developed to apply uniaxial and biaxial stretches for the controlled mechanical stimulation of cells in vitro [4,5]. Some sophisticated designs feature active micropost arrays, which can be used as actuators and/or sensors of cortical events in cells near the substrate [6]. There are also commercial devices, such as the Flexcell^®^ Tension System (Flexcell International Corp., Burlington, NC, USA), that use vacuum pressure to stretch a circular silicone membrane over a fixed loading post, and others that utilize dual motors to biaxially stretch square or rectangular wells. All these devices require substrates that allow for a homogeneous and well-defined strain distribution across the cell culture area, as well as for the application of deformation cycles [7]. Stretching experiments have greatly contributed to the basic understanding of mechanotransduction and mechanobiology, but most of them rely on devices that produce over-simplified uniaxial or equi-biaxial strain fields that may not reproduce complex in-vivo strain fields, which are often dynamic and, more importantly, multi-axial. Moreover, most devices suffer from stray deformations, which result in an additional and unwanted strain component, perpendicular to the stretching direction in uniaxial stretching experiments. This interference, often ignored, should be taken into account to rightly assess the effect of substrate strains.

None of these devices can sense cell biomechanical responses within a single cell if they are not used in combination with high-resolution techniques, such as the atomic force microscope (AFM), that can both apply and sense mechanical events at the apical membrane [2]. Herein we will show that it is possible to apply stray-free mechanical stimulations (≈10% uniaxial strain) at the basal plane and simultaneously detect apical changes in the mechanical behavior of cells down to the nanometer scale.

Dielectric elastomers are being increasingly studied as suitable materials for high-performance, all-polymeric transducers [8]. Dielectric elastomeric actuators (DEAs) are devices made of ‘smart’ materials, capable of undergoing deformations in response to suitable electrical stimuli, e.g., electrical potential. They represent an emerging class of electromechanical drives: indeed, DEAs combine high values of strain, energy density, response speed, efficiency, and resilience with low power consumption and specific weight, along with the ease of processing and low cost. Their bioinspired use has been explored to construct, for example, electrically tunable lenses as an emerging class of “artificial muscle transducers” [9,10,11]. They also hold promise for precisely controlled stretching experiments, with minimum interfering stray effects. As a matter of fact, DEAs are starting to be used as cell culture supports for cell stretching experiments [12,13]. Indeed, bioreactors that can be dynamically tunable and lead to compact, self-contained, lightweight and versatile devices, are promising for cell culture mechanical stimulation. Recently, Gao et al. have designed DEA-based bioreactors to perform mechanical stimulations (under both tensile and compressive stress) on human lung carcinoma cells for gene transfection efficiency enhancement [14]. Besides, DEAs allow many possible configurations for electrode design. This kind of versatility enables the construction of devices with electrically passive transparent zones, making it possible to combine them with optical and scanning probe microscopes.

Herein we report on a novel set-up capable of applying controlled static (i.e., constant over time) and dynamic (i.e., sinusoidal) deformations to single adherent model cells in culture at the basal membrane, via DEAs, while observing them by optical microscopy and performing AFM measurements. To test the performance of our system, we monitored the intracellular calcium changes of cardiac myocytes by fluorescence miscopy and the changes in the elastic modulus of single fibroblasts by AFM nanoindentation while applying substrate deformations. The combined set-up allowed us to monitor over time and with sub-micron spatial resolution the topography and elasticity of a single cell, in response to mechanical stimuli [15], as well its physiological activity. In particular, for the latter measurements, we monitored changes in the intracellular calcium concentration of beating cardiac myocytes. Cardiac myocytes are a rational choice for a model system: as a matter of fact, the culture of primary cardiac cells and in-situ observation of their activity is challenging under any circumstance. Moreover, it has been reported that stretching cardiac myocyte cultures has important effects in regulating sarcomere organization, hypertrophy and cell-to-cell junctions [16]. More recently, Prosser et al. [17] showed that the steady-state level of ROS production in a cardiomyocyte is graded by the amplitude and frequency with which the cell stretches. Thus, mechanical changes that depend on the pre-load and heart rate regulate a dynamic redox balance that tunes cellular Ca^2+^ signaling [18].

## 2. Materials and Methods

### 2.1. Cell Stretcher Device Working Principle

**Figure 1:**
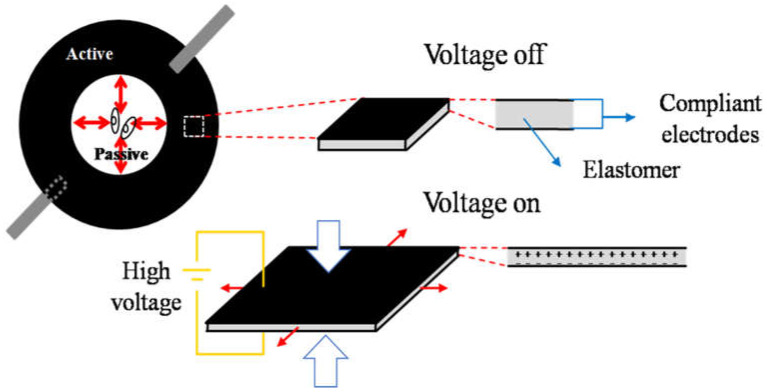
Dielectric elastomer (DE) operating principle. The radial electrode configuration distinguishes the annular (peripheral) active and circular (central) passive zones. The application of voltage across the electrodes squeezes the film between the electrodes due to the electrostatic attraction between the electrode pair. The transversal compression causes a longitudinal extension of the active zone (tensile strain). Consequently, the passive zone compresses (compressive strain).

For our cell stretcher device, we employed a thin elastomeric disk as dielectric and placed ring-shaped electrodes on both sides as depicted in Figure 1. In the following, we denominate the area covered by the electrode as the *active zone*, and the circular area in the center of the disk that is not covered by the electrodes, the *passive zone*. A voltage applied between the upper and lower electrode—i.e., across an electrode pair—generates an attractive electrostatic force that squeezes the film, reducing its thickness. This force increases with the applied voltage. Assuming that the elastomer is incompressible, the vertical stress causes a strain in the perpendicular direction with opposite sign (i.e., the film will expand laterally) [11]. As the outer diameter of the disk is clamped to a rigid structure, the active zone can only expand toward the center of the disk, thus compressing the passive zone, where cells are seeded. When the applied voltage decreases, the electrostatic force between the electrodes will decrease, their distance increase, and therefore the active zone will laterally compress, pulling the passive zone outwardly, which will stretch in turn.

### 2.2. Cell Stretcher Device Fabrication

As DEA material we employed an acrylic-polymer-based tape (VHB 4910^®^, 3M Italia, Piotello, Italy), a transparent and biocompatible film with adhesive properties. From the mechanical point of view, it behaves as an incompressible elastomer and has shown outstanding electromechanical coupling performance [19,20]. Electrodes were manually painted with a brush on both sides of the elastomer film using a conductive and compliant grease, Carbon Conductive Grease 846 (M.G Chemicals, Causeway Bay, ON, Canada).

**Figure 2:**
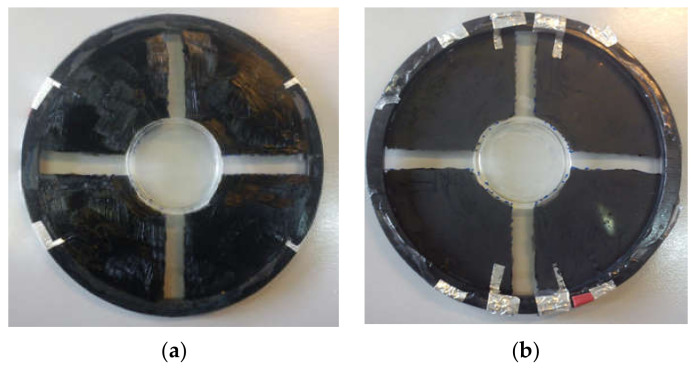
Elastomer film with four electrodes painted on each side, making a total of eight electrodes. An aluminum stripe is glued onto each electrode to make the electric contact with the driving circuit. (**a**) top view, featuring the PDMS ring glued in the center that encircles the passive zone of the device; (**b**) bottom view.

**Figure 3:**
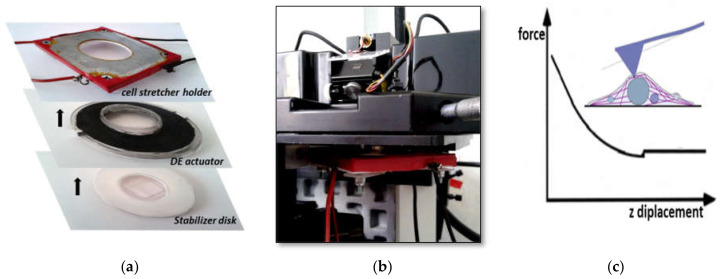
AFM-cell stretcher device system. (**a**) Pictures showing the different components of the cell stretcher device: the VHB film with the annular electrodes and the PDMS ring glued on the upper side delimiting the cell culture chamber (image in the middle). This is clamped in between the metallic holder that magnetically sticks to the AFM head (top image) and the stabilizer disk with the glass window in the center (bottom image). (**b**) Image of the cell stretcher device and the AFM head containing the probe and the scanner on top of the holder. (**c**) Schematics of the AFM tip indenting a cell and characteristic force-displacement curve (only the unloading part of the curve is plotted).

The devices consist of a circular film of elastomer, pre-stretched and fixed onto a rigid circular ring (95 mm diameter), with electrodes painted symmetrically on both sides. We verified that the film can sustain even larger deformations (we applied 300% radial pre-stretch) and behave elastically. Pre-stretching is commonly done in DEAs since it improves properties such as breakdown strength, actuation strain and efficiency [21]. Moreover, pre-stretching avoids wrinkling of the passive zone during compressive strain. After mounting the film onto the rigid frame, a soft and thin polydimethylsiloxane (PDMS) ring was cast and glued onto the upper side of the elastomer film to form the cell culture well and medium reservoir (25 mm diameter), and electrodes were manually painted using the conductive grease on both the upper and lower side. As a result, the active and passive zones became distinctive: the peripheral area covered by the electrodes where the electric field is directly applied (i.e., the active zone), and the central area, not covered by the electrodes and therefore passively strained by the expansion/contraction of the active zone (i.e., the passive zone), where cells are cultured. Thin foil aluminum stripes were glued onto the electrodes with silver paint to make the electrical contacts (Figure 2). The device was then placed on top of a rigid Teflon disk with a thin (0.18 mm) glass window in the center to allow optical access from the bottom (Figure 3a, stabilizer disk).

**Figure 4:**
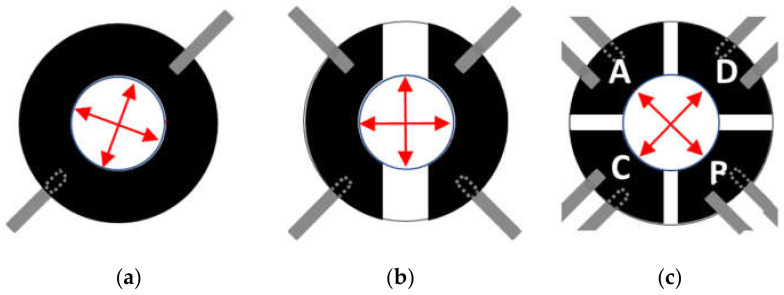
Top-view scheme of the device showing the different electrode configurations tested and the directions along which the components of the strain tensor were calculated: (**a**) annular (i.e., one pair of annular electrodes, in this case two perpendicular directions were randomly selected), (**b**) two-electrode (i.e., two pairs of partial annular electrodes); (**c**) four-electrodes (i.e., four pairs of partial annular electrodes denoted with A, B, C, and D, respectively).

To avoid the elastomer sticking onto the glass window and to reduce friction during deformations, a small drop of oil was placed onto the support before mounting the elastomer film in order to create a thin lubricant layer. The oil film significantly damped vibrations of the elastomer film, allowing AFM measurements to be performed at the required precision. Interestingly, the presence of the stabilizer disk does not significantly change the strain-voltage behavior of the device (data not shown). The device was then placed on a custom-made microscope stage that also included the AFM head (Figure 3b). Static and dynamic voltages were applied using a function generator and a high voltage amplifier module (UM8-40, Spellman High Voltage Electronics Corporation, Hauppauge, NY, USA). We tested three different electrode configurations in order to apply radial, uniaxial or biaxial strains (Figure 4a–c). In terms of electrodes pairs, a pair being two electrodes on each side of the elastomer film, the annular configuration corresponds to one electrode pair, the uniaxial configuration to two electrode pairs, and the biaxial configuration to four electrode pairs. Although voltage differences can be applied to each pair of electrodes independently, in our tests we couple the same electrical potential across electrode pairs to attain a specific deformation. In devices with the two electrode-pair configuration, the same voltage drop is applied to both electrode pairs so as to achieve uniaxial deformation, i.e., along one direction. Similarly, and in order to obtain biaxial deformations, i.e., along two mutually perpendicular directions, in devices featuring the four electrode-pair configuration, the same voltage drop is applied to two opposing electrode pairs, and zero voltage to the other two.

As expected, it was observed that the presence of the PDMS ring reduces the strain reached in the passive zone. The extent of such a decrease is higher for the radial deformation and dependent on the stiffness of the PDMS material employed (data not shown). The PDMS elastomer commonly used for biological applications is Sylgard 184 (Dow Corning Corp, Midland, MI, USA) which, in the recommended 10:1 base: crosslinker ratio, has an elastic modulus of around 2 MPa [22]. In order to reduce the stiffness of the ring, we blended Sylgard 184 with the softer PDMS gel Sylgard 527 (Dow Corning Corp, Midland, MI, USA), which has an elastic modulus of roughly 5 kPa, in the ratio 4:1 [22].

### 2.3. Strain Calibration

**Figure 5:**
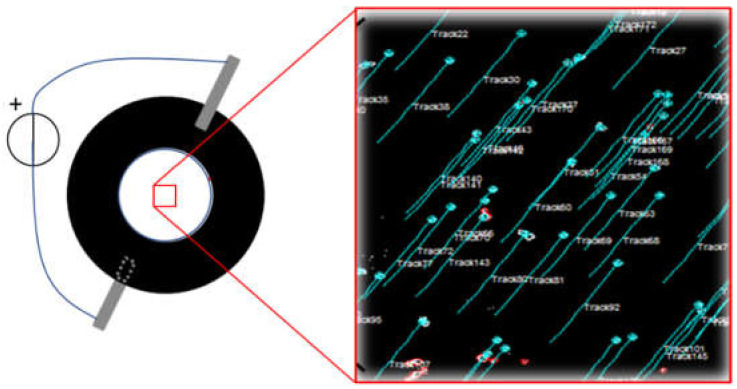
Motion tracks of fluorescent microspheres deposited on the DE film. The image shows the trajectories of single particles as a 4kV voltage step is applied.

To quantify the strain in the passive zone of the cell stretcher device, 3 µm fluorescent microbeads (excitation: 441 nm; emission: 485, Fluoresbrite^®^ YG, Polysciences Inc., Warrington, PA, USA) were used as positional markers. Microbeads were homogeneously dispersed in either water or culture medium. A 200 µL solution containing microbeads at concentration of 1.68 × 10^6^ particles/mL was deposited into the well in order to obtain, after solvent evaporation, an average surface density of 1.7 × 10^4^ particles/cm^2^, a value that allowed imaging of a large enough number of well dispersed single particles. The adhesivity of the VHB 4910 film ensured the microbeads remained stably bonded to the substrate throughout the experiment. Time-lapse sequences of fluorescence images of a 1200 × 1200 µm^2^ area were acquired with an inverted microscope in EPI fluorescence mode (IX70, Olympus, Tokyo, Japan) equipped with a CMOS camera (ORCA-Flash4.0, Hamamatsu, Hamamatsu City, Japan) while varying the voltage applied to the electrodes. Recorded sequences were then processed using ImageJ scripts to track each microbead as a function of the applied voltage (Figure 5). Approximately one hundred trajectories were extracted from each sequence and processed to determine the strain field. The strain field was calculated in terms of the components of the Green Lagrange strain tensor using a numerical tool developed in MATLAB (MathWorks, Natick, MA, USA). Briefly, the difference in position between two voltage values defines a displacement vector for each marker. The displacement data defined on the scattered cloud of points are projected on a set of points arranged on a regular grid of rectangular domains. The displacements defined on the vertex of each rectangular domain are then interpolated within the domain by using the same polynomial shape functions as those used in finite element methods. Strain components are calculated at the element Gauss points by partially differentiating the interpolating functions with respect to the spatial coordinates, according to the definition of the fully non-linear Green Lagrange strain tensor.

*Static strain calibration* as a function of voltage was obtained via the application of step-like voltages to the DEA, i.e., a fast voltage ramp from zero up to the final value, which was then kept constant. Tracking the particles displacement allowed the calculation of the static strain field for each final applied voltage. The distributions of the strain components across the passive area show normal profiles, each of which could be fully characterized by a mean and a standard deviation.

*Dynamic strain calibration* was carried out via the application of a sinusoidal voltage to the DEA at a given frequency and amplitude in the range 0.5–3 Hz and 1–4 kV, while simultaneously acquiring a sequence of images across the passive area. Off-line tracking of the particles made it possible to calculate the strain components at each particle position, which were then averaged over the imaged area for each voltage value.

### 2.4. Cell Culture

3T3 NIH Swiss mouse Fibroblast from I.Z.S.L.E.R. (Istituto Zooprofilattico Sperimentale Lombardia ed Emilia Romagna, Brescia, Italy) and cardiac myocytes (isolated from postnatal heart of Sprague-Dawley rats) were used as biological samples for the in situ AFM characterization and optical imaging of our combined setup.

Before cell plating, the surface of the well delimited by the PDMS ring was disinfected with a 70% ethanol solution, rinsed with sterile water once, and finally left under the laminar flow to dry for about 30 min. Subsequently, the well was incubated overnight with phosphate-buffered saline (PBS) solutions of gelatin (0.02%) and fibronectin (100 µg/mL) for the 3T3 cell culture, and with PBS solutions of gelatin (0.02%) and laminin (100 µg/mL) for cardiac cells. Stretching devices (without electrical connections) were placed into large petri dishes covered with Parafilm^®^ (Sigma Aldrich, St. Louis, MO, USA) to avoid evaporation. The coating solution was removed after 12 h of incubation and devices were allowed to dry under laminar flow for 15 min.

*3T3 preparation*. Cells frozen in liquid nitrogen were initially thawed and seeded on 25 cm^2^ tissue culture flasks for 24 h. Cells were maintained in culture in Dulbecco’s Modified Eagle Medium (DMEM) supplemented with fetal bovine serum (10%) and Glutamax-100 (1%), at 37 °C in a 95% humidified atmosphere and in the presence of 5% (*v/v*) CO_2_. After reaching confluence, cells were split in a 1:2 ratio using 1 mL of Trypsin–EDTA (Sigma-Aldrich C-41010, St. Louis, MO, USA). After the second passage, cells were seeded on the cell stretcher device at a lower density (9 × 10^4^ cells/cm^2^), in order to have cells sufficiently separated for AFM imaging and indentation mapping.

*Cardiomyocyte preparation*. Cryopreserved neo-natal cardiomyocytes (R-CM-561, QBM Cell Science, Ottawa, ON, Canada) [23] were thawed and immediately transferred in a conical centrifuge tube where the cell suspension was diluted with a medium composed by [24,25] DMEM-M199 (4:1), 6% Horse Serum (HS), 4% Fetal Bovine Serum (FBS), 1% Glutamax, 10 µg/mL Gentamycin. Afterwards, a cell count was performed with the hemocytometer and the single aliquots of cell suspension were plated on the devices. Two days after plating, the culture medium was replaced with Neurobasal supplemented with B27 (Gibco-Life Technologies, Waltham, MA, USA) and only 1% of horse serum to limit the overgrowth of the cardiac fibroblast cell population. The cardiomyocyte cells showed spontaneous beating within the first 24 h.

*Calcium Imaging*. To visualize the synchronous variations of the calcium signal with the rhythmic contraction of cardiac culture activity, the cardiac monolayer within the passive area was labeled with the Oregon-Green Bapta-1 AM probe (Molecular Probes-Life Technologies Cat. nr. O6807, Waltham, Massachusetts, USA). The calcium indicator powder had been reconstituted before in DMSO at 3 mM stock solution and then diluted in the medium of cardiomyocytes at the final concentration of 3 µM and incubated for 1 h at 37 °C in the dark inside the incubator. During this time, the indicator was readily hydrolized by intracellular esterase, becoming activated and responsive to calcium. At the end of this interval, the staining solution was removed from the reservoir and replaced with fresh medium without the fluorescent probe. The culture was incubated for another 40 min in the dark. Finally, the device was placed onto the microscope, equipped with a CMOS camera (ORCA-Flash4.0, Hamamatsu, Hamamatsu City, Japan), for the epifluorescence measurements at 488 nm excitation wavelength. Calcium indicator fluorescence levels were calculated from selected regions (ROIs) of video frames with ImageJ (NIH, Bethesda, MD, USA), using the well-known formula:

(1) $$\Delta F=\frac{F_{ROI}-F_{0}}{F_{0}}$$

where $F_{ROI}$ is the mean grey value of the ROI that includes the probed cells and $F_{0}$ is the background fluorescence of the frames (baseline).

### 2.5. Atomic Force Microscopy (AFM)

A commercial AFM (model 5500, Agilent Technologies, Tempe, AZ, USA) with a closed loop scanner capable of laterally scanning areas as big as 100 × 100 µm^2^ and of vertical displacements as large as 15 µm, was used to evaluate the changes in cell stiffness while strains were applied on the substrate. The instrument was mounted on top of the inverted optical microscope (model IX70, Olympus, Tokyo, Japan) equipped with the home-made stage that holds and stabilizes the cell stretcher membranes (see previous section and Figure 1). In-situ phase contrast microscopy allowed the identification of the cell before and after the application of the strain, and to position the AFM probe onto the same area. Triangular cantilevers with a sharp conical (half cone angle: 35°) tip and a nominal spring constant of 0.08 N/m (XNC12, MikroMasch, Tallinn, Estonia) were employed as probes. The actual spring constant was calculated in air before measurements as described by Hutter and Bechhoefer [26]. All force data reported here were taken using the same cantilever with a measured spring constant of 0.04 ± 0.01 N/m. Force versus distance curves were recorded at a constant speed of 5 µm/s and maximum loading force of 2.5 nN, if not stated otherwise. Complete detachment from the cell was verified between consecutive loading-unloading cycles.

**Figure 6:**
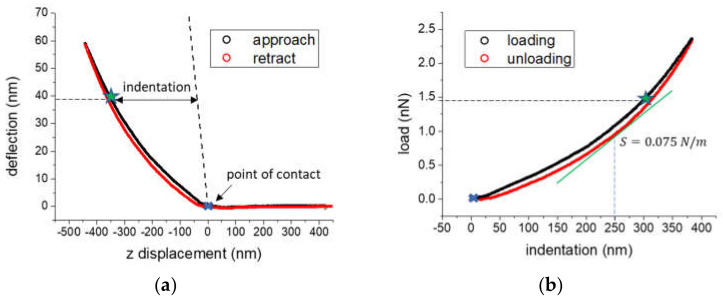
Representative force-distance curve recorded on a 3T3 cell. (**a**) Deflection as a function of the vertical position for a full approach/retract cycle; dashed line: straight line crossing the point of contact with unity slope. (**b**) Load-indentation curve calculated from the contact region of the curve plotted in (**a**). Green line: tangent to the curve at 250 nm penetration depth, its slope represents the contact stiffness S for that penetration depth.

*AFM data analysis and elastic modulus calculation*. Recorded force-distance curves were analyzed using a home-made software tool developed in LabVIEW (National Instruments, Austin, TX, USA). For each force curve, the point of tip-sample contact was first detected using a supervised automatic algorithm already described in [24]. Briefly, the far-from-contact region of the approaching part of the force curve is fitted with a straight line. The contact region, at the other end of the z displacement interval, is fitted with a quadratic function. The intersection of the two lines determines the point of contact. This is repeated for each single curve automatically, however the operator needs to approve the selections done. If not, the algorithm changes the fitting ranges in order to provide a new point of contact until it is accepted, or the curve is discarded from further analysis. Figure 6a shows a typical force curve recorded on a 3T3 cell adhered on the DE film: the cantilever deflection is plotted against the vertical (z direction) scanner displacement. The point of contact detected with the aforementioned algorithm is marked on the graph (blue cross). Deflection values can be immediately converted into force in that they are multiplied by the cantilever spring constant. The part of the force curve after contact is then converted into a load-indentation curve by calculating, for each force value, the corresponding indentation as the distance of the curve from the straight line with unity slope crossing the point of contact (Figure 6b, dashed line in the graph). The green star indicates how each point in the raw deflection vs. z displacement curve appears in the indentation curve.

We estimated the elastic modulus based on the analysis proposed by Oliver and Pharr [27] and further refined by VanLandingham and colleagues [28]. Briefly, the elastic modulus for any axis-symmetric indenter, such as the AFM conical tip, can be calculated by:

(2) $$E=\frac{1}{2}\times \sqrt{\frac{\pi }{A\left(h\right)}}\times \left(1-\nu^{2}\right)\times S\left(h\right)$$

ν is the Poisson’s ratio of the material, $S\left(h\right)=\frac{dF\left(h\right)}{dh}\;$ is the contact stiffness defined as the slope of the indentation curve $F\left(h\right)\;$ at the penetration depth *h*, and $A\left(h_{c}\right)$ represents the projected contact area at the same indentation *h*. For a conical indenter $A\left(h_{c}\right)=\pi \times { [h_{c}\times \tan\left(\alpha \right)]}^{2}$ where α is the half cone angle and $\;h_{c}$, referred as the contact depth, is related to the penetration depth by the following relation:$\;h_{c}=h-\varepsilon \times \frac{F\left(h\right)}{S\left(h\right)}$ where ε is a dimensionless coefficient dependent on the tip geometry.

We used a value of ν=0.5, assuming the cell as incompressible, and of ε=0.75, assuming a conical tip geometry, and considered the load and the contact stiffness at a penetration depth h=250 nm.

As mentioned above, the elasticity values calculated and obtained via the AFM technique are commonly used estimations of the true mechanical response of the cell. Indeed, the technique introduces few experimental uncertainties, related to the determination of the contact point, approximations on the tip geometry, and the projected contact area between tip and cell; on the other hand, the model above neglects cell viscoelasticity, anisotropy, and heterogeneity which certainly play a role when indenting cells at relatively high speeds as is usually done in AFM measurements. These limitations, though important, are out of the scope of this paper, whose focus is on the operability of the DEA-based device and the combined experimental setup.

*Measurements of cell elasticity at different deformations*. Force measurements were performed on a regular 8 × 8 grid on the same single fibroblast, across a 500 × 500 nm^2^ area, at different times during the compressed and extended states.

High resolution cell elasticity maps were also acquired on 3T3 cells in order to verify the stability of the cell stretcher setup and visualize the effects of the applied mechanical stimulus across larger areas. In these experiments, force measurements were performed on 64 × 64 positions across a 40 × 40 µm^2^ area of the same cell before and after the application of the mechanical stimulus.

## 3. Results and Discussion

### 3.1. Cell Stretcher Device Performance

#### 3.1.1. Static Strain Response

**Figure 7:**
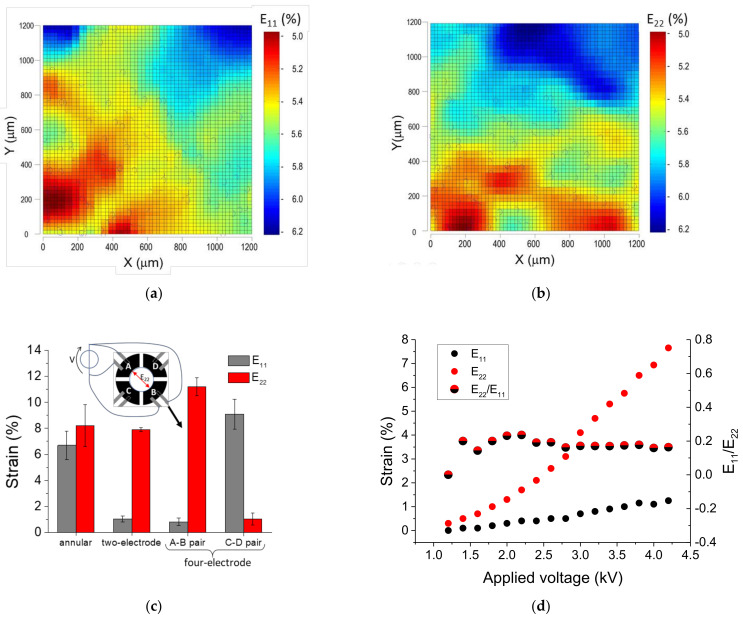
Static characterization of the deformation. (**a**,**b**) typical strain fields for a device with annular configuration, the blue circles represent the initial position of the tracking beads; (**c**) maximum strains (average and standard deviation) obtained with devices in the annular, two- and four-electrode configurations when the voltage V is applied either to the A–B pairs or to the C–D pairs; (**d**) strain performance of a device in the two-electrode configuration.

Figure 7a,b show a typical result of the deformation field across an area of 1.2 × 1.2 mm^2^ in the center of the passive zone of a device with an annular configuration, and after the application of a voltage step (4000 V → 0 V). The color maps report the strain field in terms of the components of the Green Lagrange strain tensor, E_11_ (Figure 7a) and E_22_ (Figure 7b). The blues circles report the initial positions of the beads whose trajectory was used to calculate the strain.

Figure 7c (bar diagram) shows the strain along the two Cartesian directions (E_11_ and E_22_) for different electrode configurations: annular, two-electrode pairs, and four-electrode pairs. For the latter, the results are shown when the voltage is applied to the electrode pair along E_22_ or the other one, respectively. Data are average values and standard deviations of five consecutive 4000 V → 0 V voltage steps calculated over randomly selected areas around the center of the passive zone, which is the area reachable by the AFM probe.

For the annular configuration, the average values of E_11_ and E_22_ across the whole region correspond to tensile strains (i.e., stretching) of 6.7 ± 1.1% and 8.2 ± 1.2% (average ± std), respectively. These results and the radial symmetry of the annular electrode configuration allow the application of fairly similar strains along two randomly selected and mutually perpendicular directions, hence achieving a state of equi-biaxial deformation.

Figure 7d shows the strain vs. applied voltage curve of a device with a two-electrode configuration. The graph shows that the strain along the longitudinal direction with respect to the electrodes (E_22_) depends rather linearly on the applied voltage, and that the strain in the perpendicular direction (E_11_) is comparatively small. It can also be observed that the ratio E_22_/E_11_ does not significantly depend on the applied voltage. Such a behavior makes the application of controlled uniaxial deformations along a specific direction with minimal stray field interference possible.

#### 3.1.2. Dynamic Strain Response

**Figure 8:**
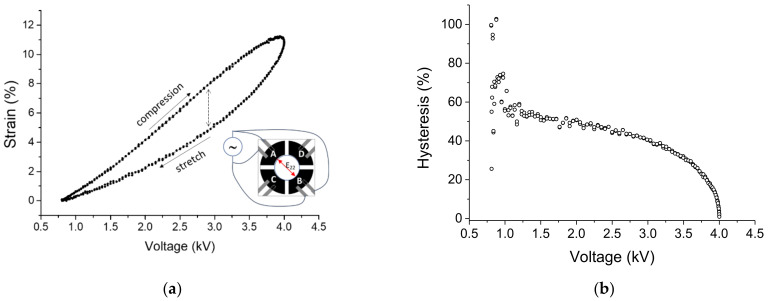
Dynamic characterization of the deformation for a device with a four-electrode configuration. (**a**) Strain component E_22_ along the direction of the active electrode pairs A and B is plotted as a function of a sinusoidal voltage with 0.5 Hz frequency for three different cycles, dotted arrow indicates the maximum absolute hysteresis in the cycle. (**b**) Corresponding compression/stretch strain ratio as a function of the applied voltage.

Figure 8 shows the strain response for a two electrode-pair device when driven 0.5 Hz sinusoidal voltage in the range 0.8 kV–4 kV. Data were obtained through a continuous signal acquisition for three successive cycles. Figure 8a shows E_11_ deformation component averaged over a 1.2 × 1.2 mm^2^ area in the center of the passive zone. It can be observed that the deformation of the device follows the applied voltage signal with high repeatability (we observed the same strain response up to an hour of continuous dynamic actuation) and with a maximum absolute strain difference of 2.3% due to hysteresis. The relative hysteresis, calculated as the normalized difference between the strains during compression and stretching, is plotted in Figure 8b as a function of the applied voltage.

The observed strain response is of comparable magnitude to that reported for DEA-based electro-actuated devices [13] and is significantly more sensitive than uniaxial stretchers driven by step motors or pneumatic systems. Indeed, the device can produce and control uniform strains below 10%, with a stray field of approximately 9–13% of the maximum applied strain, in both its two-electrode and four-electrode pair configurations. As shown in Figure 7d, the signal to noise ratio, defined by the quotient between the strain (E_11_) and stray (E_22_) fields, increases with the strain field. Contrarily, devices on stretchable membranes operate at higher uniaxial strains, 20–50%, with a perpendicular component of 15–20% [29]. In addition, the possibility to apply fairly biaxial strains in virtually any set of perpendicular directions, without the introduction of additional mechanical elements or actuators, places the proposed cell stretcher device in a strongly advantageous position with respect to multiaxial strain devices, which require multiple step motors, complex and noisy mechanisms to achieve the same result [5]. In addition, both uniaxial and biaxial devices, mechanically and pneumatically driven, have limited control and capacity to generate uniform strain fields [5], whereas devices based on actuated micropost arrays are conceived for the application of very local, subcellular stresses, and do not have such a capacity. The fairly homogeneous and controllable strain levels attained through the electro-actuated device herein presented adds to its outperformance and versatility. Indeed, the one-stop capacity of this device makes it suitable to investigate different cell types, which predominantly sustain unidirectional (vascular) or multidirectional (connective) stress in vivo.

#### 3.1.3. Optical Performance and Biocompatibility

We tested the devices with different cell types and herein report the results obtained with primary cardiac cells and 3T3 fibroblasts. After seeding, cells were allowed to grow and spread on the cell stretcher device for few days in an incubator. In all cases cells thrived, enabling time-lapse fluorescence microscopy experiments with primary cardiac cells and AFM mechanical measurements with 3T3 fibroblasts (see next section).

**Figure 9:**
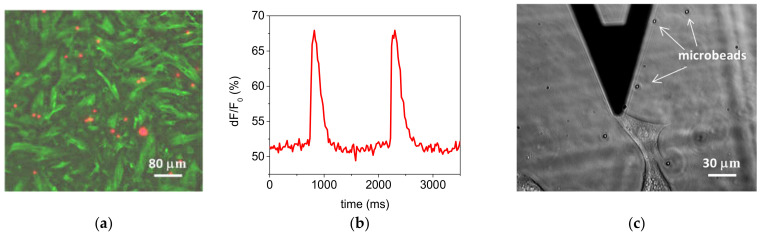
Optical performance of the combined setup. (**a**) Fluorescent micrograph of adherent cardiac myocytes cultured on the cell stretcher device; (**b**) beating cycle reconstructed from time-lapse fluorescence micrographs showing time-dependent calcium flux variations and (**c**) phase contrast micrograph of a 3T3 fibroblast and few microbeads (dark triangular object is the AFM cantilever).

We set out to test the optical performance of our device and the mechanical behavior of cardiac myocytes attached to DE substrates (Figure 9a) by in-situ monitoring the flux of Ca^2+^ across cell membranes as a function of time. Time-lapse fluorescence micrographs were acquired during myocyte beating activity, which captured the changes in fluorescence inside the cells as Ca^2+^ concentration changes. The myocyte beating cycle could be reconstructed from time series of images by integrating the fluorescence signal over the area of single cells, as exemplified in Figure 9b. The DE film optical transparency enabled the optical visualization of cells cultured onto the DE film during AFM operation, and the (re)positioning of the AFM tip on the same cell area after the application of deformations that can cause large sample displacements. Figure 9c shows an exemplary image.

### 3.2. Cell Elasticity Response to Strain

**Figure 10:**
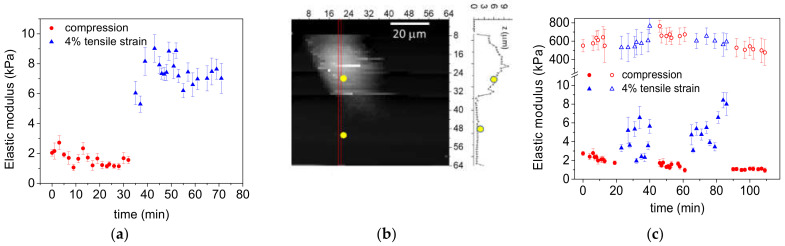
Elastic modulus of fibroblasts subjected to step-like voltage changes as a function of time, before and after uniaxial compression-stretching (4% strain). Each elasticity data point shows the mean value and standard deviation calculated from 8 × 8 indentation curves across an area of 500 × 500 nm^2^. (**a**) Cell elastic modulus during one compression-stretching cycle. (**b**) Topography image of a single 3T3 cell obtained from a 64 × 64 force map and height profile corresponding to the red line. The yellow dots correspond to the two areas probed to calculate the elasticity values reported in (**c**). (**c**) Two and a half consecutive compression/stretch cycles. The elasticity values measured on the bare dielectric elastomer are also plotted for comparison (empty symbols).

The cell elasticity of single 3T3 cells cultured on the device substrate has been studied with AFM as a response to strain. Figure 10a shows the results of an experiment in which a series of 8 × 8 force-distance curve maps were recorded while static uniaxial 4% strains have been applied to the substrate. The average and standard deviation of the elasticity values of every single map are plotted as a function of the recording time. An increase in cell elastic modulus (i.e., in cell stiffness) can be clearly observed while the cell is stretched.

In order to check if the observed change in stiffness is reproducible, reversible, and characteristic of the cell rather than the substrate or of the measurement procedure, we first calculated the topography of the cell (Figure 10b) in the stretched state and then the elasticity on top of the cell and on the substrate (Figure 10b, yellow dots) for repeated deformations. Particular care was taken to repeat the measurements on the same spot (500 × 500 nm^2^ area) of the same cell over time and at different deformations. The probed area on the cell was chosen in such a way that it was not too close to the cell nucleus nor to the cell edges in order to avoid nucleus and substrate effects in the AFM indentation measurements. As a rule of thumb often employed in indentation tests, when the thickness of the sample is at least ten times the max indentation depth, one can neglect the effect of a hard substrate on the measured mechanical property of the sample. The height profile corresponding to the yellow dot in Figure 10b corresponds to a cell thickness of more than 5 µm, twenty times the penetration depth used for the calculation of the elasticity value.

Figure 10c shows the elasticity values corresponding to the two yellow spots marked in the height profile: a net increase in cell stiffness (Figure 10c, filled symbols) can be observed after each uniaxial stretch that reverses to the initial elasticity values during the compression. Contrarily, (Figure 10c, empty symbols) the substrate does not exhibit changes in elastic modulus under the same applied strains.

The results demonstrate that the fibroblast senses and responds to substrate deformations and that it significantly stiffens when it senses tensile strains. Since cell stiffening (response) is probed on top of the cells while being mechanically stimulated at the bottom, the observed change in elasticity provides evidence of an active cellular response.

**Figure 11:**
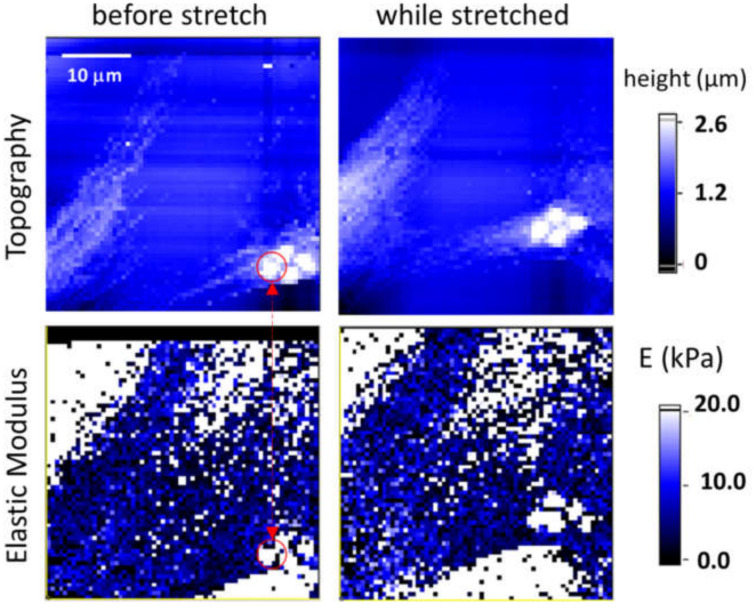
Topography and elasticity maps of a 3T3 fibroblast before (**left**) and during (**right**) mechanical stimulation. Scanning area is 40 × 40 µm^2^. A cluster of four microspheres serves as a positional marker to identify the same cell. Elastic modulus is reported only in the cellular region (black color otherwise).

High-resolution AFM force mapping is a lengthy type of experiment that demands high and long-term stability in terms of low instrumental drift and vibrations. The stretching device proved suitable for this kind of essays, as evinced by Figure 11. The figure shows topography and elasticity maps of a single fibroblast, before and during 4% uniaxial strain; each map entails the acquisition and analysis of 64 × 64 force curves taken over 40 × 40 µm^2^ area. The topography is calculated as the piezo extension at the maximum applied force of 2 nN, while the elastic modulus is calculated at 250 nm indentation depth. Although a fast vertical speed was applied for each force curve (20 µm/s), it took almost 45 min to acquire a single map. After the first mapping, the electrode voltage was switched from 2500 V to 1200 Volt in order to apply a static tensile strain of 4%. The strain was calculated from the positions of the fluorescent beads which had been deposited in the culture medium before the experiment. A four-bead cluster was used as positional marker to perform the experiment on the same area after the application of the strain, which causes an overall lateral shift. As shown in Figure 11, the setup enabled obtaining high resolution images on roughly the same region within a single cell despite the duration of the experiment. This indicates that the system is sufficiently stable (i.e., very low creep effects) to perform high-demanding experiment. As expected, the elastic modulus averaged over the mapped cell area increase when the cell is stretched, from 6.96 kPa to 11.15 kPa, considerably higher than those reported in Figure 10. One should mention that these measurements, unlike the previous ones, have been taken on the peripheral area of the cell, where the cell thickness is lower and, therefore, the underlying stiff substrate can influence the calculated elastic modulus. However, we can ascribe the observed increase in stiffness to a cellular effect, as, according to the previous observations, only cells, and not the substrate, respond to a mechanical deformation, hence opening the way for the investigation of mechanisms of basal-to-apical force transmission in cells. Previous results on changes in cell elasticity measured by AFM as a response to an applied static stretch are somehow mixed: a softening of epithelial cells as a response to a 20% tensile strain [29] and a stiffening of keratinocytes subjected to strains ≥25% [30]. These results cannot be directly compared to the stiffening of 3T3 fibroblast that we observed for much smaller strains.

## 4. Conclusions

Herein we have presented a combined instrumental setup that allows the controlled application of uniaxial and biaxial, static and dynamic strains across a dielectric elastomer, and the simultaneous examination of the morphology and the mechanical behavior of cells as they are mechanically stimulated. The device can apply, in a controlled way, “large” (≈12% strain) directional deformations at up to 2 Hz cycling speeds and is suitable for the long-term culture of primary cells, stem cells and standard cell lines, as well as high-resolution microscopy. Although we have not yet performed a systematic characterization of the stability of the applied strain over cyclic deformations and long durations, we have not noticed any material fatigue effect. The most critical aspect for long-term operation during cell culture in a humid environment such as an incubator is water condensation over the electrodes and the consequent occurrence of shortcuts. The possibility of applying cyclic deformations is fundamental: cell reorientation, the most visible effect of mechanical stretching, is often a consequence of the substantial remodeling of the actin cytoskeleton as a cellular response. This response is directly correlated with changes in the mechanical properties of the cells [30], and enhanced by oscillating deformations of the substrate, especially at low frequencies (<1 Hz) [31]. Thus, the option of cyclic stimulation in combination with optical microscopy and AFM enables, in principle, the investigation of the coupling between mechanical response and cytoskeletal remodeling, in situ and at the micro- and nanoscale.

The proof-of-performance experiments on 3T3 fibroblasts also reveal cellular stiffening upon the application of 4% tensile strains on the substrate that reverses when the strain is removed and the film compressed.

Few studies on the cellular response to substrate deformation have been already presented, yet our combined setup constitutes a significant advancement in this respect. The work of Tremblay et al. demonstrated the applicability of stand-alone stretcher devices, which allowed the application of uniaxial and biaxial deformations on substrates onto which the morphological response of epithelial cells could be studied [32]. On the other hand, few cell stretcher systems based on soft PDMS elastomer films have been proposed and designed in order to be compatible with AFM and optical microscopy operation and apply uniaxial static large deformations [29,33]. Our combined set-up goes a step beyond the state of the art in that it enables the application, in a controlled way, not only of static, but also dynamic strains along two mutually perpendicular directions and, potentially, along more directions using a multielectrode configuration. This enables the study of cells under biaxial strains and quantitative elasticity measurements with high sensitivity by AFM nanoindentation. These types of studies have been scarce, despite the fact that biaxial loading conditions are common in cells of certain tissues (e.g., the pericardium) [7].

## Data Availability

Data sharing is not applicable to this article.
